# Genetic polymorphisms of metabolic enzyme genes associated with leukocyte mitochondrial DNA copy number in PAHs exposure workers

**DOI:** 10.1002/cnr2.1361

**Published:** 2021-03-31

**Authors:** Xinling Li, Xiaoran Duan, Hui Zhang, Mingcui Ding, Yanbin Wang, Yongli Yang, Wu Yao, Xiaoshan Zhou, Wei Wang

**Affiliations:** ^1^ Department of Occupational Health and Occupational Diseases, College of Public Health Zhengzhou University Zhengzhou China; ^2^ The Key Laboratory of Nanomedicine and Health Inspection of Zhengzhou Zhengzhou China; ^3^ Safety Management Department of Anyang Iron and Steel Group Company Anyang China; ^4^ Department of Epidemiology and Biostatistics, College of Public Health Zhengzhou University Zhengzhou China

**Keywords:** 1‐OHPYR, COEs, gene polymorphisms, *GSTP1* rs1695, mtDNAcn

## Abstract

**Background:**

Polycyclic aromatic hydrocarbons (PAHs) exposure had been reported to be a risk factor of mtDNAcn in our early study. However, the effect of metabolic enzymes' genetic polymorphisms on mtDNAcn in PAHs‐Exposure workers has not been fully evaluated.

**Aim:**

The aim of the study was to explore the effect of metabolic enzymes' genetic polymorphisms on mtDNAcn in PAHs‐Exposure.

**Methods and Results:**

We investigated the effects of metabolic enzymes' genetic polymorphisms on mtDNAcn among 544 coke oven workers and 238 office staffs. The mtDNAcn of peripheral blood leukocytes was measured using the Real‐time quantitative polymerase chain reaction (PCR) method. PCR and restriction fragment length was used to detect five polymorphisms in *GSTT1*, *GSTM1*, *GSTP1* rs1695, *CYP2E1* rs6413432, and *CYP2E1* rs3813867. The mtDNAcn in peripheral blood leukocytes was significantly lower in the exposure group than that in the control group (*p* < .001). The 1‐OHPYR had an increasing trend with the genotypes AA→AG → GG of *GSTP1* rs1695 in the control group. Generalized linear model indicated that the influencing factors of mtDNAcn were PAHs‐exposure [*β* (95% CI) = −0.420 (−0.469, −0.372), *p* < .001], male [*β* (95% CI) = −0.058 (−0.103, −0.012), *p* = .013], and AA genotype for *GSTP1* rs1695 [*β* (95% CI) = −0.051 (−0.095, −0.008), *p* = .020].

**Conclusion:**

The individuals carrying the AA genotype of *GSTP1* rs1695 may have a lower mtDNAcn due to their weaker detoxification of PAHs.

## 1 INTRODUCTION

Coke oven emissions (COEs) are generally derived from the incomplete combustion of organic matter and possess hazardous levels of fine particulate and polycyclic aromatic hydrocarbons (PAHs). Most chemicals of PAHs are classified as human carcinogens and the genotoxic potential of PAHs has been extensively studied. In addition to producing damage on the nuclear DNA,^1^ PAHs have 40 to 90‐fold higher affinity for mitochondrial DNA (mtDNA) than nuclear DNA and cause even higher level damage to mtDNA.^2^ Several studies have found that PAHs may interfere mitochondrial biosynthesis and further alter mitochondrial DNA copy number (mtDNAcn).^3^, ^4^, ^5^ Our previous study also found that mtDNAcn decreased with environmental PAHs‐exposure.^6^

Mitochondria play an important role in multiple cellular functions including oxidative phosphorylation, reactive oxygen species generation, calcium homeostasis, and apoptosis. Each mitochondrial contains 2–10 copies of mitochondrial DNA. Due to the lack of protective histones and DNA repair machinery, mtDNA is particularly more vulnerable to various kinds of environmental toxins.^7^ The mtDNAcn will increase as compensation to early poison exposure,^8^ however, when the increased mtDNAcn cannot maintain the mitochondria normal function, mitophagy will occur to remove dysfunctional ones, and the mtDNAcn will be reduced.^9^ Therefore, mtDNAcn might be a sensitive and important target to the genotoxic of PAHs. Moreover, the alteration of mtDNAcn has also been found to be associated with various disease development. For example, alterations of mtDNAcn are interrelated with lung cancer risk^10^, and the decreased mtDNAcn may accelerate the aging process and causes age‐related disorders.^11^

After PAHs enter the human body, they are firstly metabolized to electrophilic active intermediates by phase Imetabolic enzymes, such as cytochrome P450 (*CYP*) monooxygenases. The metabolic intermediates may produce DNA adducts, leading to DNA mutations, alteration of gene expression, and even tumorigenesis.^12^ Subsequently, the intermediates are converted into more polar and water‐soluble products by phase II metabolic enzymes and then excreted from the body.^13^ The phase II metabolic enzymes include glutathione S‐transferases (*GST*), *UDP* glucuronyl transferases, *NADPH* quinone oxidoreductases, aldo‐keto reductases, and epoxide hydrolases. Therefore, extensive polymorphism of metabolism enzymes genes may be one of the main reasons for the individual variable susceptibility to exogenous toxic substances.^14^ Studies have shown that metabolic enzymes' genetic polymorphisms were associated with many health effects in PAHs exposure population. Whyatt et al. found that the *CYP1A1* Mspl restriction site had higher DNA adduct levels among newborns with PAHs exposure.^15^ One study suggested that the *GSTM1* (−) was inversely associated with the DNA integrity in the men occupationally exposed to PAHs.^16^ Our earlier study showed that *GSTT1* (+) and *GSTM1* (+) are the risk factors for oxidative stress in coke oven workers.^17^ However, the influence of metabolic enzymes' genetic polymorphisms on mtDNAcn in PAHs‐Exposure workers has not been studied yet.

Therefore, we detected mtDNAcn to investigate the genotoxic of PAHs‐exposure and screened *GSTT1*, *GSTM1*, *GSTP1*, and *CYP2E1* gene to explore the role of metabolic enzymes genetic polymorphisms on mtDNAcn in PAHs‐exposure.

## 2 MATERIALS AND METHODS

### 2.1 Study population and epidemiological data

A random cluster sampling method was adopted to enroll the subjects in this study. A total of 544 workers exposed to COEs for more than 1 year were recruited as the exposure group from the Henan Anyang Iron and Steel Group, Henan, China. Their workplaces were in five representative locations, including auxiliary production, office personnel, oven bottom, oven side, and oven top. Also, 238 healthy workers without a history of exposure to occupational poison were enrolled from the same region as the control group. Exposure‐group subjects were selected based on the following inclusion criteria: (a) age from 18 to 60, (b) who worked in the coke plant and were occupationally exposed to COEs for more than 1 year, and (c) provided informed consent. Subjects were excluded if they had major organ function failure or a tumor, were pregnant, or were lactating. Except for the history of occupational exposure, the inclusion and exclusion criteria for the control group were similar to those in the exposure group.

Detailed information on general demographic characteristics, professional history, and biological samples from each participant was collected by trained interviewers. The blood was collected using Na_2_EDTA and heparin anticoagulants, and the urine (the end‐of‐work urine of the occupational exposure population and morning urine of the control group) was retained with 50 mL centrifuge tubes. The study protocol and consent form from all subjects were subjected to approval by the Ethics Committee of Zhengzhou University, China. More detailed information is described in our previous study.^6^

### 2.2 Determination of environmental exposure

According to the Sampling specifications for monitoring hazardous substances in the workplace air (GBZ159‐2004) and Exhaust for the stable pollution source‐Determination of benzene soluble matter‐Soxhlet extraction (HJ690‐2014), the representative air samples were collected with a medium flow sample and the COE cumulative exposure dose of the exposure group were determined. Based on living environmental concentration and age, the COE cumulative exposure dose of the control group was estimated. Detailed detection methods were described in our previous study.^18^

High‐performance liquid chromatography (HPLC) was used to detect the concentrations of four OH‐PAHs [1‐hydroxypyrene (1‐OHPYR) and 1‐hydroxynathalene (1‐OHNAP), 2‐hydroxynathalene (2‐OHNAP), and 3‐hydroxyphenanthrene (3‐OHPHE)] in urine, as described in the previous study.^19^

### 2.3 Analysis of mtDNAcn

DNA was extracted from the peripheral blood leukocytes using a Large Amount of Whole Blood Genomic DNA Extraction Kit (Beijing BioTeke Corporation). The mtDNAcn was measured using the Real‐time quantitative polymerase chain reaction (PCR) method. This assay measures relative mtDNAcn by determining the ratio of the ND‐1 mitochondrial gene to the human β‐globin gene. The ND‐1 mitochondrial gene primers were forward, 5′‐CCTAATGCTT ACCGAACGA −3′ and reverse, 5′‐G GGTGATGGTAGATGTGGC‐3′. The β‐globin gene was forward, 5′‐GCTTCTGACACAACTGTGT TCACTAGC‐3′ and reverse, 5′‐CACCAACTTC ATCCACGTTCACC‐3′. Detailed information as described previously.^6^

### 2.4 Detection of genetic polymorphisms

**FIGURE 1:**
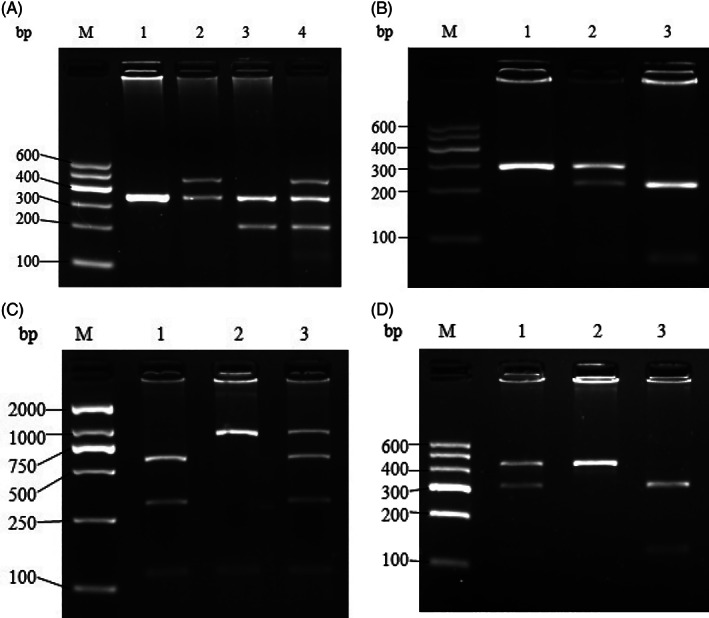
(A) PCR products of *GSTT1* and *GSTM1*. M: DNA marker; lanes 1‐4 DNA products; lane 1: *GSTT1*(−) and *GSTM1*(−); lane 2: *GSTT1*(+) and *GSTM1*(−); lane 3: *GSTT1*(−) and *GSTM1*(+); lanes 4: *GSTT1*(+) and *GSTM1*(+). (B) PCR‐RLFP products of *GSTP1* rs1695. M: DNA marker; lanes 1‐3 DNA products; lane 1: AA; lane 2: AG; lane 3: GG. (C) PCR‐RLFP products of *CYP2E1* rs6413432. M: DNA marker; lanes 1‐3 DNA products; lane 1: AA; lane 2: TT; lane 3: AT. (D) PCR‐RLFP products of *CYP2E1* rs3813867. M: DNA marker; lanes 1‐3 DNA products; lane 1: GC; lane 2: GG; lane 3: CC

The *GSTT1* and *GSTM1* genotype were determined as previously described.^20^ PCR‐restriction fragment length polymorphism (PCR‐RFLP) method was used to detect other loci for genotyping, including *GSTP1* rs1695, *CYP2E1* rs6413432, and *CYP2E1* rs3813867.^21^ The primers are shown in Table 1. To ensure the accuracy of the experiment, the positive control, and the negative control were set both in the process of PCR and restriction enzyme digestion. What's more, 10 % of each gene polymorphic loci samples were selected randomly for repeat and the concordance was 100%. The representative gel pictures for PCR‐RFLP were shown in Figure 1.

**TABLE 1:** Primers sequences for gene polymorphism

Polymorphism		Primers sequences
*GSTT1*	Forward:	5′‐TTCCTTACTGGTCCTCACATCTC‐3′
	Reverse:	5′‐TCACCGGATCATGGCCAGCA‐3′
*GSTM1*	Forward:	5′‐GAACTCCCTGAAAAGCTAAAG C‐3′
	Reverse:	5′‐GTTGGGCTCAAATATACGGTG‐3′
*ALB* a	Forward:	5′‐GCCCTCTGCTAACAAGTCCTAC‐3′
	Reverse:	5′‐GCCCTAAAAAGAAAATCGCCAATC‐3′
*GSTP1* rs1695	Forward:	5′‐CTTCCACGCACATCCTCTTCC‐3′
	Reverse:	5′‐AAGCCCCTTTCTTTGTTCAGC‐3′
*CYP2E1* rs6413432	Forward:	5′‐TCGTCAGTTCCTGAAAGCAGG‐3′
	Reverse:	5′‐GAGCTCTGATGCAAGTATCGCA‐3′
*CYP2E1* rs3813867	Forward:	5′‐CCAGTCGAGTCTACATTGTCA‐3′
	Reverse:	5′‐TTCATTCTGTCTTCTAACTGG‐3′

*Note*: Forward represents the upstream primer and reverse represents the downstream primer.

a *ALB* is a control gene used in multiple PCR for *GSTT1* and *GSTM1*.

### 2.5 Statistical analysis

Baseline survey data were entered using EpiData 3.1 software. All analyses were performed using SPSS 25.0 software (SPSS, Inc., Chicago, Illinois). The data of four OH‐PAHs were converted by natural logarithm to satisfy normal distribution. After adjusting appropriate adjustments for gender, age (years), smoking status, drinking status, and BMI, covariance analysis was used to analyze the effects of the general characteristics and gene polymorphism on OH‐PAHs or mtDNAcn. Multiple linear regression analyzed the trend of OH‐PAHs change with mutant allele loci. The generalized linear model (GLM) analyzed the influencing factors of mtDNAcn by adjusting the smoking index, drinking status, and BMI. All statistical tests were two‐sided, and the level of statistical significance was set at *α* = .05.

## 3 RESULTS

### 3.1 Individual characteristics and mtDNAcn

The cohort consists of 544 COEs‐exposed workers as the exposure group, and 238 healthy people. The proportion of male, smoking, and drinking in the exposure group (71.7, 41.0, and 54.4%) were higher than that in the control group (58.4, 17.2, and 42.0%; *p* < .05). The age in the exposure group (40.10 ± 6.30) was significantly older than that in the control group (38.39 ± 8.43; *p* = .005). The BMI had no significant difference between the exposure group and the control group (*p* = .376). The COEs cumulative exposure dose and the four OH‐PAHs in the exposure group were higher than those in the control group (*p* < .05). MtDNAcn in peripheral blood leukocytes was significantly lower in the exposure group (0.60 ± 0.29) than that in the control group (1.03 ± 0.31; *t* = 18.931, *p* < .001). The basic characteristics have been reported in our previous research^22^ (Table 2).

**TABLE 2:** The general characteristics of control and exposure groups

Variables^a^	Control	Exposure	*χ*^2^*/t*/*Z*	*p*
Gender				
Male	139 (58.4)	390 (71.7)	13.357^b^	<.001
Female	99 (41.6)	154 (28.3)		
Age grouping				
≤40	142 (59.7)	273 (50.2)	5.974^b^	.015
>40	96 (40.3)	271 (49.8)		
Age (years)	38.39 ± 8.43	40.10 ± 6.30	2.800^c^	.005
Smoking status				
No	197 (82.8)	321 (59.0)	41.817^b^	<.001
Yes	41 (17.2)	223 (41.0)		
Drinking status				
No	138 (58.0)	248 (45.6)	10.176^b^	.001
Yes	100 (42.0)	296 (54.4)		
BMI (kg/m^2^)				
Low weight	5 (2.1)	12 (2.2)	3.101^b^	.376
Normal weight	108 (45.4)	224 (41.2)		
Overweight	101 (42.4)	230 (42.3)		
Obesity	24 (10.1)	78 (14.3)		
Cumulative exposure dose	0.07 (0.06,0.09)	1.12 (0.34, 2.14)	22.093^d^	<.001
1‐OHPYR	1.78 ± 0.98	4.44 ± 1.15	19.953 ^a^	<.001
1‐OHNAP	3.02 ± 0.92	4.08 ± 1.20	3.151 ^a^	.002
2‐OHNAP	3.31 ± 0.10	4.49 ± 1.03	9.792 ^a^	<.001
3‐OHPHE	0.99 ± 1.12	2.96 ± 1.06	16.828 ^a^	<.001
mtDNAcn	1.03 ± 0.31	0.60 ± 0.29	18.931 ^a^	<.001

^a^ Each variable is represented by the number of samples and percentage or mean ± *SD*.

^b^ *χ*^2^ test.

^c^ *t*‐test.

^d^ Rank sum test.

### 3.2 The effects of gender, age, smoking, drinking, and BMI on mtDNAcn

After adjusting appropriate adjustments of gender, age (years), smoking index, drinking status, and BMI, covariance analysis showed that no factors were related to the mtDNAcn (*p* > .05). However, the mtDNAcn of all layers had significant differences between the two groups (*p* < .001). As reported in our early study,^6^ the results are shown in Table 3.

**TABLE 3:** The effect of general characteristics on mtDNAcn

Variable	Control		Exposure		*F*	*p*
	*n*	mtDNAcn ($\bar{x}\pm s$)	*n*	mtDNAcn ($\bar{x}\pm s$)		
Gender						
Male	139	1.00 ± 0.28	390	0.59 ± 0.30	201.939	<.001
Female	99	1.08 ± 0.34	154	0.62 ± 0.29	133.108	<.001
*F*		3.085		0.231		
*P*		0.080		0.631		
Age grouping						
≤40	142	1.05 ± 0.31	273	0.60 ± 0.28	223.722	<.001
>40	96	1.01 ± 0.30	271	0.60 ± 0.31	114.037	<.001
*F*		0.816		0.062		
*P*		0.367		0.804		
Smoking status						
No	197	1.04 ± 0.32	321	0.60 ± 0.29	257.887	<.001
Yes	41	0.99 ± 0.27	223	0.59 ± 0.29	64.900	<.001
*F*		0.030		0.201		
*P*		0.863		0.654		
Drinking status						
No	138	1.05 ± 0.33	248	0.61 ± 0.30	168.387	<.001
Yes	100	1.01 ± 0.27	296	0.58 ± 0.29	164.549	<.001
*F*		0.065		0.650		
*P*		0.800		0.420		
BMI (kg/m^2^)						
<24.0	113	1.01 ± 0.33	236	0.62 ± 0.30	115.357	<.001
24.0‐27.9	101	1.06 ± 0.29	230	0.58 ± 0.28	194.732	<.001
≥28	24	1.03 ± 0.30	78	0.58 ± 0.33	32.382	<.001
*F*		1.530		0.783		
*p*		.219		.458		

*Note*: Covariance analysis was used to analyze the effects of the general characteristics on mtDNAcn with the appropriate adjustment of gender, age (years), smoking index, drinking status, and BMI.

### 3.3 Effects of genetic polymorphisms on 1‐OHPYR

The genotype distribution for each genetic polymorphism locus did not deviate from the Hardy‐Weinberg balance (*p* > .05; Table 4), and the allele frequencies were similar to those of Asians in the International Human Genome HapMap Project, suggesting the control samples had representativeness.

**TABLE 4:** Distribution of SNP loci and Hardy–Weinberg equilibrium test

Gene/SNPs	Genotype	Number	Genotype Frequency	Allele Frequency	Hardy–Weinberg *χ* ^2^ (*P*)^a^
*GSTT1*	−	101	0.424	−	−
	+	137	0.576		
*GSTM1*	−	130	0.546	−	−
	+	108	0.454		
*GSTP1* rs1695					
	AA	148	0.622	A: 0.790	0.039 (0.844)
	AG	80	0.336	G: 0.210	
	GG	10	0.042		
*CYP2E1* rs6413432					
	TT	144	0.605	T: 0.780	0.048 (0.827)
	AT	83	0.349	A: 0.220	
	AA	11	0.046		
*CYP2E1* rs3813867					
	GG	133	0.559	G: 0.742	0.509 (0.476)
	CG	87	0.366	C: 0.258	
	CC	18	0.076		

^a^ The Hardy–Weinberg test was performed using the control group.

The differences in 1‐OHPYR among metabolic enzyme genes polymorphisms are shown in Table 5. With the adjustment of the covariates affecting 1‐OHPYR, covariance analysis showed that the 1‐OHPYR in non‐deletion for *GSTM1* was significantly higher than that in deletion in the exposure group (*p* = .024), the 1‐OHPYR in AA for *GSTP1* rs1695 was significantly higher than that in AG genotype in the control group (*p* = .044). After adjusting the covariates (gender, age, smoking index, drinking status, and BMI), the trend test of multiple linear regression revealed that the 1‐OHPYR had an increasing trend with the genotypes AA → AG → GG in the control group (*p* = .013).

**TABLE 5:** The effect of metabolic enzyme gene polymorphism on 1‐OHPYR

Gene/SNPs	Control			Exposure		
	*n*	1‐OHPYR ($\bar{x}\pm s$)	*p*	*n*	1‐OHPYR ($\bar{x}\pm s$)	*p*
*GSTT1*						
−	35	1.79 ± 0.83	Ref	150	4.49 ± 1.09	Ref
+	53	1.76 ± 1.08	.768	205	4.44 ± 1.20	.936
*GSTM1*						
−	51	1.71 ± 0.77	Ref	205	4.32 ± 1.10	Ref
+	37	1.86 ± 1.22	.452	150	4.61 ± 1.21	.024
*GSTP1* rs1695						
AA	53	1.62 ± 0.89	Ref	239	4.41 ± 1.11	Ref
AG	32	1.95 ± 1.07	.044	107	4.46 ± 1.26	.582
GG	3	2.66 ± 1.04	.077	9	5.17 ± 1.04	.059
*P*_trend_		0.013			0.160	
*CYP2E1* rs6413432						
TT	53	1.63 ± 0.82	Ref	210	4.50 ± 1.11	Ref
AT	32	2.00 ± 1.21	.260	127	4.29 ± 1.22	.067
AA	3	1.87 ± 0.37	.774	18	4.82 ± 1.16	.120
*P*_trend_		0.307			0.747	
*CYP2E1* rs3813867						
GG	49	1.64 ± 0.76	Ref	216	4.41 ± 1.21	Ref
CG	36	1.89 ± 1.20	.473	119	4.53 ± 1.01	.761
CC	3	2.60 ± 1.09	0.187	20	4.32 ± 1.37	.517
*P*_trend_		0.219			0.830	

*Note*: Covariance analysis was used to compare 1‐OHPYR among genotypes, adjusted for gender, age (years), smoking index, drinking status, and BMI. Multiple linear regression analyzed the trend of 1‐OHPYR change with mutant allele loci, adjusting gender, age, smoking index, drinking status, BMI. Ref: The reference group when comparing.

### 3.4 Effects of genetic polymorphisms on mtDNAcn

As shown in Table 6, there were no statistically significant differences in mtDNAcn among different genotypes in loci of the metabolic enzyme genes. The mtDNAcn in AG + GG for *GSTP1* rs1695 was slightly higher than that in the AA genotype in the exposure group (*p* = .077).

**TABLE 6:** The effect of metabolic enzyme gene polymorphism on mtDNAcn

SNPs	Control			Exposure		
	*n*	mtDNAcn ($\bar{x}\pm s$)	*p*	*n*	mtDNAcn ($\bar{x}\pm s$)	*p*
*GSTT1*						
−	101	1.03 ± 0.33	Ref	236	0.57 ± 0.30	Ref
+	137	1.04 ± 0.30	.969	308	0.61 ± 0.29	.113
*GSTM1*						
−	130	1.04 ± 0.29	Ref	313	0.60 ± 0.30	Ref
+	108	1.02 ± 0.33	.689	231	0.60 ± 0.28	.929
*GSTP1* rs1695						
AA	148	1.02 ± 0.29	Ref	361	0.58 ± 0.29	Ref
AG	80	1.04 ± 0.33	.585	172	0.63 ± 0.31	.062
GG	10	1.17 ± 0.38	.057	11	0.56 ± 0.36	.917
*P*_trend_		0.132			0.126	
*GSTP1* rs1695						
AA	148	1.02 ± 0.29	Ref	361	0.58 ± 0.29	Ref
AG + GG	90	1.06 ± 0.34	.308	183	0.63 ± 0.31	.077
*CYP2E1* rs6413432						
TT	144	1.04 ± 0.31	Ref	315	0.60 ± 0.29	Ref
AT	83	1.03 ± 0.31	.867	199	0.59 ± 0.30	.748
AA	11	0.96 ± 0.32	.568	30	0.57 ± 0.27	.578
*P*_trend_		0.828			0.573	
*CYP2E1* rs3813867						
GG	133	1.01 ± 0.29	Ref	340	0.61 ± 0.30	Ref
CG	87	1.07 ± 0.33	.231	167	0.59 ± 0.28	.644
CC	18	1.06 ± 0.34	.546	37	0.52 ± 0.29	.112
*P*_trend_		0.261			0.168	

*Note*: Covariance analysis was used to compare mtDNAcn among genotypes, adjusted for gender, age (years), smoking index, drinking status, and BMI. Multiple linear regression analyzed the trend of mtDNAcn change with mutant allele loci, adjusting gender, age, smoking index, drinking status, BMI. Ref: The reference group when comparing.

### 3.5 The influencing factors on mtDNAcn

The influencing factors were screened by GLMs with mtDNAcn as the dependent variable, PAHs exposure, gender, age, *GSTT1*, *GSTM1*, *GSTP1* rs1695, *CYP2E1* rs6413432, and *CYP2E1* rs3813867 gene as predicators, and smoking status, drinking status, and BMI as covariates. The variables kept in the model included PAHs‐exposure (*b* = −0.436, *p* < .001), male (*b* = −0.058, *p* = .013) and genotype AA for *GSTP1* rs1695 (*b* = −0.051, *p* = .020) (Table 7).

**TABLE 7:** The influencing factors of mtDNAcn

Influencing factors	*β* (95% CI)	*χ* ^2^	*p*
Constant	1.552 (1.221, 1.884)	84.278	<.001
PAHs‐exposure	−0.420 (−0.469, −0.372)	289.770	<.001
Male	−0.058 (−0.103, −0.012)	6.127	.013
*GSTP1* rs1695 AA	−0.051 (−0. 095, −0.008)	5.395	.020

*Note*: GLMs was used to analyze the influencing factors of mtDNAcn adjusted for smoking index, drinking status, and BMI.

Abbreviation: PAHs, polycyclic aromatic hydrocarbons.

## 4 DISCUSSION

Workers in coke oven plants are exposed to a wide variety of volatile organic compounds and particulates, especially PAHs. The PAHs and their metabolic intermediates might cause damage to the mitochondria.^23^ Increasing evidence indicates that PAHs‐exposure may relate to mtDNAcn decrease. Ling et al. observed that decreased sperm mtDNAcn was associated with PAHs‐exposure in the male population in Chongqing, China.^5^ Pieters et al. demonstrated that mtDNAcn was inversely associated with indoor PAHs exposure population and their findings were also confirmed in human TK6 cells.^3^ Our previous study showed that mtDNAcn had significantly negative correlations with the levels of COE cumulative exposure dose.^24^ Moreover, our previous study also found that mtDNAcn had significantly negative correlations with the levels of 1‐OHPYR which can be used to estimate the internal exposure of PAHs. Though the covariance analysis showed the mtDNAcn was not significantly different in gender, the male had lower mtDNAcn than the female in the controls and exposure group. What's more, the GLM indicated that the male may be a risk factor of the decreased mtDNAcn [*β* (95% CI) = −0.058 (−0.103, −0.012), *p* = .013]. Vyas et al.^25^ found that female has higher mtDNAcn compared to male in a diverse cohort of mid‐life and older adults. Yu et al.^26^ observed the same gender‐specific phenomenon in a cross‐sectional analysis. The gender difference in mtDNAcn may be due to the mitochondrial maternal inheritance, which enables females to better maintain mitochondrial functions and control over mtDNAcn.^27^

*GSTT1*, *GSTM1*, and *GSTP1* are the important phase II metabolic enzymes in glutathione‐*S*‐transferases enzymes system and the genetic polymorphisms in these enzyme genes may alter gene expression levels and its enzymes activity, and subsequently, involve toxicity of PAHs.

The *GSTP1* rs1695 is located on exon 5 of chromosome 11q13, and contains a wild‐type G allele and mutant A allele. The transition of an A allele to G allele in *GSTP1* rs1695 confers increased conjugating activity^28^, ^29^ and may also with lower levels of genotoxicity in PAHs exposure. Our study found that the 1‐OHPYR had an increasing trend with the genotypes AA → AG → GG of *GSTP1* rs1695 in the control group. Moreover, our results firstly showed that AA for *GSTP1* rs1695 was a risk factor for mtDNAcn in PAHs‐exposure. Therefore, we inferred that the toxicity of PAHs may be influenced by *GSTP1* rs1695 polymorphisms resulting in the different alteration of mtDNAcn.

Though we also analyzed the association between mtDNAcn and polymorphisms in *GSTT1*, *GSTM1*, there was no significant difference. The possible reason is that mitochondrial copy number is affected by many factors, and the polymorphism of the metabolic enzyme gene has a modest effect on mtDNAcn. However, our result showed that the 2‐OHNAP and 3‐OHPHE in non‐deletion for *GSTM1* were significantly higher than that in deletion in the exposure group. This confirms that metabolic enzyme gene polymorphism may alter the toxicity of PAHs.

*CYP2E1* is an important member of *CYP450* system and is located on *Homo sapiens* chromosome 10q24.3. The *CYP2E1* rs3813867 is a G/C polymorphism located at 1259 position in the 5′‐flanking region and the *CYP2E1* rs6413432 (T > A) located on intron 6, which can affect the *CYP2E1* gene expression level and its enzyme activity.^30^ Therefore, polymorphism of *CYP2E1* may affect the activity of the enzyme express leading to individual differences in PAHs metabolism. Nan et al. observed that *CYP2E1* polymorphism was an important factor influencing the levels of 1‐OHPYR and 2‐OHNAP in urinary from coke oven workers.^31^ In this study, there was no significant difference between OH‐PAHs and *CYP2E1* polymorphism, that may be because the PAHs are mostly activated by *CYP1A* and *CYP1B*.^32^

Polymorphism of *CYP2E1* may also influence the genotoxic of environmental toxins. Guang et al.^33^ found that the *CYP2E1* rs3813867 mutant allele was associated with higher micronuclei among benzene‐exposed shoe workers. Jheneffer et al.^34^ reported that alcoholics who heterozygous in the *CYP2E1* rs3813867 showed higher DNA damage (tail length and olive tail moment). Jing et al.^35^found heterozygous in the *CYP2E1* rs6413432 had shorter telomere lengths among benzene‐exposed shoe workers. However, mtDNAcn change was not associated with *CYP2E1* polymorphism in the present study.

In the study, we have analyzed a larger number of samples, which is the advantage of the study. However, several limitations of the present study need to be considered. First, due to the cross‐sectional design of this study, a causal relationship between PAHs exposure and mitochondria damage cannot be established. Second, White blood cell differentials and platelet concentrations, which are the major sources of mtDNAcn variability, were not assessed. Third, a large number of studies have shown that there are differences in metabolic enzyme gene polymorphism among different populations. The effects of metabolic enzyme gene polymorphism on mtDNAcn of coke oven workers may not be suitable for all world populations in this study. Forth, increasing studies have proved that particulate matter exposure may alter mitochondrial dynamics leading to the change of mtDNAcn. In 63 male healthy steel workers, Hou et al.^36^ found that particulate matter exposure was positively associated with mtDNAcn. However, Hou et al.^37^ observed decreased mtDNAcn in association with increased exposure to particulate matter in a repeated‐measure study. Wong et al.^38^ and Wang et al.^39^ also found that particulate matter exposure was linked with decreased mtDNAcn. Therefore, the relationship between particulate matter exposure and mtDNAcn needs to be further studied. Due to lack of data on particulate matter exposure, we did not analysis the effect of particulate matter on mtDNAcn and we would consider it in further study. Hence, further research is needed to address these questions.

In conclusion, the individuals carrying the AA genotype of *GSTP1* rs1695 may have a lower mtDNAcn due to their weaker detoxification of PAHs.

## AUTHOR CONTRIBUTIONS

**Xinling Li:** Formal analysis; writing‐original draft. **Xiaoran Duan:** Investigation. **Hui Zhang:** Formal analysis. **Yanbin Wang:** Investigation. **Xiaoshan Zhou:** Formal analysis; writing‐review and editing. **wei wang:** Formal analysis; funding acquisition; writing‐review and editing.

## CONFLICT OF INTEREST

The authors declare there is no conflict of interest.

## ETHICAL STATEMENT

All procedures performed in studies involving human participants were following the ethical standards of the institutional and or national research committee and with the 1964 Helsinki Declaration and its later amendments or comparable ethical standards. The study protocol and consent form from all subjects were subjected to approval by the Ethics Committee of Zhengzhou University, China.

## Data Availability

The data will be available if requested from the corresponding author.
